# Supplementary material for: A Likelihood Approach for Real-Time Calibration of Stochastic Compartmental Epidemic Models
Source: PLoS Comput Biol. 2017 Jan 17;13(1):e1005257. doi: 10.1371/journal.pcbi.1005257 (PMC5240920; doi:10.1371/journal.pcbi.1005257)
Supplement: S1 File — (TAR.GZ) [file pcbi.1005257.s014.tar.gz › HSPH_Online-SI-Revision/PF08/n100-severe/n100-severe_PF2_table4.pdf]

1	newly 3 weeks spec	1675.	3687.17	{3511., 3858.}	120.13	0.342366	newly 3 weeks cumul	7166.	13 161.6	{12 653., 13 711.}	83.6679	0.263786	newly 5 weeks spec	875.	2535.94	{2391., 2673.}	189.822	0.461778	newly 5 weeks cumul	9240.	18 771.3	{18 048., 19 534.}	103.153	0.30758	newly 46 weeks cumul	11 305.	29 211.6	{27 932., 30 434.}	158.395	0.412025
2	newly 3 weeks spec	260.	3347.95	{3260., 3437.}	1187.67	1.10972	newly 3 weeks cumul	1782.	13 310.8	{13 115., 13 512.}	646.96	0.873267	newly 5 weeks spec	65.	1930.5	{1870., 1983.}	2870.	1.47265	newly 5 weeks cumul	1949.	17 805.3	{17 498., 18 035.}	813.56	0.960711	newly 53 weeks cumul	2047.	23 680.8	{23 248., 23 995.}	1056.86	1.06325
3	newly 3 weeks spec	220.	3673.06	{3603., 3745.}	1569.57	1.22255	newly 3 weeks cumul	1526.	14 542.8	{14 417., 14 663.}	853.004	0.979086	newly 5 weeks spec	41.	2119.39	{2059., 2173.}	5069.24	1.71333	newly 5 weeks cumul	1670.	19 459.4	{19 361., 19 562.}	1065.23	1.06641	newly 54 weeks cumul	1741.	25 861.5	{25 727., 25 968.}	1385.44	1.17185
4	newly 3 weeks spec	1102.	3421.05	{3299., 3555.}	210.44	0.491821	newly 3 weeks cumul	5564.	13 395.4	{13 115., 13 661.}	140.752	0.38151	newly 5 weeks spec	463.	2015.26	{1936., 2099.}	335.261	0.638527	newly 5 weeks cumul	6701.	18 048.1	{17 660., 18 506.}	169.335	0.430224	newly 52 weeks cumul	7579.	24 370.5	{23 805., 25 200.}	221.554	0.507155
5	newly 3 weeks spec	2014.	3225.5	{3109., 3333.}	60.1539	0.204388	newly 3 weeks cumul	7895.	11 594.	{11 265., 11 873.}	46.8527	0.166801	newly 5 weeks spec	1193.	2192.01	{2083., 2295.}	83.7393	0.263906	newly 5 weeks cumul	10 658.	16 471.8	{15 974., 16 936.}	54.5487	0.188973	newly 46 weeks cumul	14 294.	25 518.4	{24 360., 26 376.}	78.5252	0.251512
6	newly 3 weeks spec	1306.	3929.03	{3806., 4026.}	200.845	0.478256	newly 3 weeks cumul	6189.	15 056.	{14 775., 15 337.}	143.27	0.386044	newly 5 weeks spec	562.	2395.65	{2292., 2479.}	326.272	0.629526	newly 5 weeks cumul	7603.	20 516.1	{20 043., 20 901.}	169.842	0.431059	newly 52 weeks cumul	8616.	28 268.	{27 575., 28 855.}	228.087	0.515925
7	newly 3 weeks spec	678.	3730.06	{3641., 3810.}	450.156	0.740419	newly 3 weeks cumul	3914.	14 686.9	{14 505., 14 882.}	275.24	0.574287	newly 5 weeks spec	239.	2176.59	{2115., 2253.}	810.707	0.959262	newly 5 weeks cumul	4550.	19 724.8	{19 476., 19 994.}	333.512	0.636981	newly 53 weeks cumul	4820.	26 346.4	{25 972., 26 758.}	446.606	0.73765
8	newly 3 weeks spec	1197.	3828.33	{3719., 3912.}	219.827	0.504782	newly 3 weeks cumul	5691.	14 082.3	{13 881., 14 237.}	147.449	0.393451	newly 5 weeks spec	560.	2452.57	{2358., 2544.}	337.959	0.641223	newly 5 weeks cumul	7047.	19 607.3	{19 292., 19 835.}	178.236	0.444364	newly 49 weeks cumul	8398.	28 273.6	{27 674., 28 809.}	236.671	0.527098
9	newly 3 weeks spec	121.	5351.21	{5252., 5446.}	4322.49	1.64563	newly 3 weeks cumul	1328.	20 348.	{20 230., 20 466.}	1432.23	1.18532	newly 5 weeks spec	4.	3209.1	{3137., 3273.}	80 127.5	2.90426	newly 5 weeks cumul	1373.	27 729.	{27 612., 27 849.}	1919.59	1.30526	newly 54 weeks cumul	1373.	37 757.9	{37 639., 37 860.}	2650.03	1.43934
10	newly 3 weeks spec	840.	4690.13	{4600., 4772.}	458.349	0.746864	newly 3 weeks cumul	4139.	15 150.	{15 015., 15 272.}	266.031	0.563508	newly 5 weeks spec	240.	3744.03	{3655., 3824.}	1460.01	1.19305	newly 5 weeks cumul	4837.	23 131.5	{22 969., 23 289.}	378.22	0.679621	newly 47 weeks cumul	5228.	42 978.2	{42 687., 43 239.}	722.078	0.914907
11	newly 3 weeks spec	467.	3586.49	{3488., 3686.}	667.985	0.885234	newly 3 weeks cumul	2925.	14 224.1	{14 027., 14 513.}	386.294	0.686862	newly 5 weeks spec	130.	2069.86	{2004., 2137.}	1492.2	1.20188	newly 5 weeks cumul	3288.	19 027.8	{18 788., 19 360.}	478.704	0.762426	newly 53 weeks cumul	3458.	25 266.5	{24 961., 25 759.}	630.668	0.863689
12	newly 3 weeks spec	1748.	4259.47	{4146., 4353.}	143.677	0.386749	newly 3 weeks cumul	7573.	14 224.4	{14 074., 14 351.}	87.8303	0.273755	newly 5 weeks spec	887.	3217.03	{3148., 3291.}	262.687	0.559465	newly 5 weeks cumul	9747.	21 160.1	{21 029., 21 296.}	117.093	0.33664	newly 46 weeks cumul	12 152.	36 962.3	{36 709., 37 233.}	204.166	0.483103
13	newly 3 weeks spec	1801.	3383.3	{3224., 3606.}	87.8567	0.273433	newly 3 weeks cumul	7188.	12 173.8	{11 726., 12 980.}	69.3634	0.228551	newly 5 weeks spec	954.	2294.15	{2173., 2434.}	140.477	0.380637	newly 5 weeks cumul	9478.	17 279.7	{16 591., 18 412.}	82.3137	0.26054	newly 46 weeks cumul	11 854.	26 615.7	{25 472., 28 690.}	124.529	0.350915
14	newly 3 weeks spec	2332.	3802.7	{3703., 3909.}	63.066	0.21227	newly 3 weeks cumul	8946.	13 245.3	{13 013., 13 453.}	48.0585	0.1704	newly 5 weeks spec	1422.	2709.58	{2631., 2792.}	90.5471	0.279877	newly 5 weeks cumul	12 296.	19 183.9	{18 888., 19 433.}	56.0177	0.193147	newly 46 weeks cumul	16 703.	31 048.	{30 402., 31 550.}	85.8828	0.269201
15	newly 3 weeks spec	957.	4595.12	{4514., 4669.}	380.159	0.681347	newly 3 weeks cumul	4781.	16 034.7	{15 884., 16 168.}	235.384	0.525532	newly 5 weeks spec	410.	3223.57	{3137., 3306.}	686.237	0.895463	newly 5 weeks cumul	5823.	23 135.8	{22 977., 23 309.}	297.318	0.599132	newly 48 weeks cumul	6577.	36 547.	{36 261., 36 815.}	455.679	0.744816
16	newly 3 weeks spec	2249.	4469.44	{4376., 4549.}	98.7301	0.298201	newly 3 weeks cumul	9354.	15 501.2	{15 381., 15 653.}	65.7169	0.219338	newly 5 weeks spec	1205.	3170.32	{3083., 3254.}	163.097	0.420028	newly 5 weeks cumul	12 257.	22 470.1	{22 307., 22 653.}	83.3246	0.263199	newly 47 weeks cumul	15 533.	36 054.8	{35 784., 36 301.}	132.117	0.365697
17	newly 3 weeks spec	539.	4177.73	{4085., 4257.}	675.089	0.889268	newly 3 weeks cumul	2954.	16 237.1	{16 032., 16 438.}	449.665	0.740067	newly 5 weeks spec	198.	2472.94	{2398., 2558.}	1148.96	1.0964	newly 5 weeks cumul	3479.	21 930.8	{21 651., 22 218.}	530.376	0.799566	newly 53 weeks cumul	3686.	29 566.5	{29 185., 29 990.}	702.131	0.904202
18	newly 3 weeks spec	789.	3765.79	{3644., 3888.}	377.286	0.678619	newly 3 weeks cumul	4078.	14 577.5	{14 325., 14 875.}	257.467	0.553152	newly 5 weeks spec	352.	2185.28	{2078., 2269.}	520.818	0.792593	newly 5 weeks cumul	4968.	19 634.3	{19 255., 19 915.}	295.215	0.596735	newly 51 weeks cumul	5544.	25 865.6	{25 272., 26 242.}	366.551	0.668763
19	newly 3 weeks spec	457.	3096.51	{3010., 3181.}	577.573	0.830844	newly 3 weeks cumul	2528.	12 139.9	{11 966., 12 405.}	380.218	0.6814	newly 5 weeks spec	174.	1830.91	{1771., 1885.}	952.247	1.02195	newly 5 weeks cumul	2969.	16 362.6	{16 142., 16 684.}	451.114	0.741201	newly 51 weeks cumul	3262.	22 113.3	{21 782., 22 563.}	577.905	0.831124
20	newly 3 weeks spec	674.	4554.46	{4483., 4641.}	575.736	0.829736	newly 3 weeks cumul	3591.	16 203.6	{16 096., 16 327.}	351.229	0.654391	newly 5 weeks spec	266.	3079.7	{3011., 3149.}	1057.78	1.06356	newly 5 weeks cumul	4252.	23 037.6	{22 906., 23 149.}	441.807	0.733841	newly 49 weeks cumul	4608.	35 026.	{34 812., 35 233.}	660.113	0.880874
21	newly 3 weeks spec	521.	3748.22	{3646., 3819.}	619.428	0.856919	newly 3 weeks cumul	3210.	14 744.1	{14 559., 14 968.}	359.318	0.662092	newly 5 weeks spec	157.	2182.92	{2117., 2262.}	1290.39	1.14301	newly 5 weeks cumul	3675.	19 791.5	{19 564., 20 035.}	438.544	0.731202	newly 53 weeks cumul	3774.	26 432.3	{26 161., 26 759.}	600.379	0.845315
22	newly 3 weeks spec	558.	5314.61	{5178., 5428.}	852.439	0.978764	newly 3 weeks cumul	3543.	18 633.8	{18 353., 18 834.}	425.932	0.720909	newly 5 weeks spec	178.	3586.79	{3464., 3702.}	1915.05	1.30417	newly 5 weeks cumul	4026.	26 638.5	{26 223., 26 903.}	561.662	0.820615	newly 51 weeks cumul	4274.	39 665.5	{38 803., 40 307.}	828.065	0.96753
23	newly 3 weeks spec	35.	4334.14	{4247., 4401.}	12 283.3	2.09279	newly 3 weeks cumul	468.	17 016.5	{16 890., 17 152.}	3536.	1.56062	newly 5 weeks spec	5.	2529.75	{2472., 2590.}	50 495.	2.70403	newly 5 weeks cumul	490.	22 872.9	{22 740., 23 015.}	4567.95	1.66912	newly 55 weeks cumul	497.	30 504.3	{30 386., 30 642.}	6037.69	1.788
24	newly 3 weeks spec	330.	4512.35	{4428., 4591.}	1267.38	1.13585	newly 3 weeks cumul	2438.	17 613.8	{17 525., 17 711.}	622.468	0.858814	newly 5 weeks spec	72.	2646.57	{2575., 2702.}	3575.79	1.56528	newly 5 weeks cumul	2664.	23 737.9	{23 637., 23 843.}	791.062	0.949905	newly 56 weeks cumul	2695.	31 836.1	{31 733., 31 956.}	1081.3	1.07236
25	newly 3 weeks spec	192.	4566.24	{4480., 4656.}	2278.25	1.37621	newly 3 weeks cumul	1247.	17 752.1	{17 536., 18 004.}	1323.58	1.15336	newly 5 weeks spec	31.	2699.69	{2632., 2761.}	8608.68	1.93989	newly 5 weeks cumul	1365.	23 984.6	{23 755., 24 284.}	1657.11	1.24479	newly 56 weeks cumul	1367.	32 232.2	{32 029., 32 504.}	2257.88	1.37251
26	newly 3 weeks spec	830.	4717.06	{4634., 4790.}	468.32	0.754551	newly 3 weeks cumul	4614.	16 751.2	{16 645., 16 852.}	263.05	0.559961	newly 5 weeks spec	289.	3136.89	{3070., 3206.}	985.429	1.03555	newly 5 weeks cumul	5390.	23 764.6	{23 625., 23 904.}	340.901	0.644337	newly 50 weeks cumul	5805.	35 308.5	{35 053., 35 595.}	508.243	0.78407
27	newly 3 weeks spec	578.	3961.99	{3886., 4056.}	585.465	0.835925	newly 3 weeks cumul	3242.	15 213.5	{15 084., 15 346.}	369.262	0.671405	newly 5 weeks spec	170.	2392.18	{2327., 2452.}	1307.16	1.14824	newly 5 weeks cumul	3757.	20 682.9	{20 514., 20 847.}	450.515	0.740761	newly 51 weeks cumul	4031.	28 375.4	{28 088., 28 655.}	603.93	0.847517
28	newly 3 weeks spec	1075.	3204.29	{3096., 3334.}	198.073	0.474177	newly 3 weeks cumul	4677.	12 261.	{12 067., 12 618.}	162.154	0.418498	newly 5 weeks spec	430.	1907.03	{1804., 1996.}	343.495	0.646561	newly 5 weeks cumul	5845.	16 661.8	{16 349., 17 185.}	185.061	0.454869	newly 50 weeks cumul	7034.	22 476.1	{22 019., 23 204.}	219.535	0.504424
29	newly 3 weeks spec	909.	3224.6	{3122., 3357.}	254.741	0.549747	newly 3 weeks cumul	4464.	12 688.	{12 394., 12 971.}	184.23	0.453599	newly 5 weeks spec	364.	1898.38	{1841., 1962.}	421.533	0.717103	newly 5 weeks cumul	5336.	17 074.1	{16 687., 17 436.}	219.979	0.505051	newly 51 weeks cumul	5817.	22 981.3	{22 303., 23 593.}	295.071	0.596582
30	newly 3 weeks spec	1036.	4160.22	{4063., 4246.}	301.566	0.603698	newly 3 weeks cumul	4857.	14 158.9	{14 044., 14 281.}	191.516	0.464653	newly 5 weeks spec	473.	3035.27	{2974., 3117.}	541.706	0.807254	newly 5 weeks cumul	6087.	20 773.7	{20 618., 20 898.}	241.28	0.533105	newly 47 weeks cumul	7069.	34 707.2	{34 475., 34 975.}	390.978	0.691055
31	newly 3 weeks spec	35.	4537.74	{4462., 4625.}	12 865.	2.11273	newly 3 weeks cumul	499.	17 657.9	{17 548., 17 751.}	3438.65	1.54883	newly 5 weeks spec	17.	2666.56	{2604., 2737.}	15 585.6	2.19542	newly 5 weeks cumul	538.	23 820.5	{23 696., 23 920.}	4327.61	1.64617	newly 56 weeks cumul	540.	31 952.3	{31 832., 32 062.}	5817.09	1.77211
32	newly 3 weeks spec	307.	4308.82	{4219., 4392.}	1303.52	1.14717	newly 3 weeks cumul	2113.	16 782.2	{16 684., 16 903.}	694.235	0.899943	newly 5 weeks spec	67.	2526.94	{2468., 2594.}	3671.55	1.57643	newly 5 weeks cumul	2352.	22 623.6	{22 504., 22 733.}	861.886	0.98312	newly 54 weeks cumul	2475.	30 381.1	{30 245., 30 507.}	1127.52	1.08903
33	newly 3 weeks spec	386.	3404.91	{3315., 3485.}	782.101	0.945447	newly 3 weeks cumul	2219.	13 600.1	{13 496., 13 739.}	512.894	0.787376	newly 5 weeks spec	106.	1958.74	{1896., 2014.}	1747.87	1.26656	newly 5 weeks cumul	2533.	18 159.6	{18 061., 18 279.}	616.922	0.855467	newly 54 weeks cumul	2695.	24 055.9	{23 960., 24 191.}	792.614	0.95066
34	newly 3 weeks spec	1133.	4571.15	{4470., 4654.}	303.455	0.605745	newly 3 weeks cumul	5334.	14 637.	{14 513., 14 759.}	174.41	0.43839	newly 5 weeks spec	504.	3685.9	{3613., 3757.}	631.329	0.864056	newly 5 weeks cumul	6608.	22 460.5	{22 274., 22 621.}	239.899	0.531342	newly 46 weeks cumul	7621.	42 661.2	{42 378., 42 968.}	459.784	0.748014
35	newly 3 weeks spec	193.	4810.41	{4721., 4892.}	2392.44	1.39658	newly 3 weeks cumul	1207.	18 654.4	{18 519., 18 786.}	1445.52	1.18907	newly 5 weeks spec	21.	2827.46	{2757., 2897.}	13 364.1	2.1291	newly 5 weeks cumul	1310.	25 191.8	{25 036., 25 337.}	1823.04	1.28398	newly 55 weeks cumul	1313.	33 831.3	{33 691., 33 969.}	2476.64	1.41105
36	newly 3 weeks spec	618.	4429.68	{4347., 4508.}	616.777	0.85534	newly 3 weeks cumul	3733.	17 166.9	{17 052., 17 294.}	359.869	0.662627	newly 5 weeks spec	195.	2613.03	{2552., 2675.}	1240.02	1.12704	newly 5 weeks cumul	4250.	23 191.	{23 063., 23 344.}	445.67	0.736925	newly 53 weeks cumul	4446.	31 242.	{31 067., 31 480.}	602.7	0.846765
37	newly 3 weeks spec	114.	4041.54	{3968., 4113.}	3445.21	1.54961	newly 3 weeks cumul	920.	15 840.	{15 737., 15 936.}	1621.74	1.23596	newly 5 weeks spec	26.	2354.61	{2291., 2418.}	8956.19	1.95684	newly 5 weeks cumul	1039.	21 277.5	{21 167., 21 393.}	1947.88	1.3113	newly 55 weeks cumul	1048.	28 413.7	{28 280., 28 540.}	2611.23	1.43316
38	newly 3 weeks spec	477.	3574.21	{3495., 3643.}	649.31	0.874608	newly 3 weeks cumul	2807.	14 189.4	{14 075., 14 283.}	405.501	0.703715	newly 5 weeks spec	136.	2055.58	{2000., 2110.}	1411.46	1.1793	newly 5 weeks cumul	3197.	18 972.3	{18 885., 19 067.}	493.441	0.773374	newly 53 weeks cumul	3359.	25 158.4	{25 062., 25 260.}	648.986	0.874471
39	newly 3 weeks spec	862.	3341.69	{3194., 3468.}	287.667	0.588257	newly 3 weeks cumul	4078.	12 702.3	{12 422., 13 001.}	211.483	0.493363	newly 5 weeks spec	352.	2018.97	{1896., 2132.}	473.571	0.758125	newly 5 weeks cumul	4972.	17 338.3	{16 825., 17 826.}	248.718	0.542368	newly 50 weeks cumul	5595.	23 569.6	{22 621., 24 455.}	321.261	0.624351
40	newly 3 weeks spec	607.	3912.3	{3729., 4036.}	544.53	0.808973	newly 3 weeks cumul	3292.	15 260.4	{14 795., 15 565.}	363.559	0.665983	newly 5 weeks spec	210.	2242.46	{2142., 2319.}	967.838	1.02808	newly 5 weeks cumul	3890.	20 472.4	{19 782., 20 845.}	426.284	0.721054	newly 53 weeks cumul	4289.	26 896.4	{25 742., 27 416.}	527.101	0.797045
41	newly 3 weeks spec	231.	3863.67	{3775., 3936.}	1572.58	1.22334	newly 3 weeks cumul	1656.	15 284.3	{15 189., 15 388.}	822.964	0.965178	newly 5 weeks spec	65.	2235.81	{2164., 2294.}	3339.71	1.5364	newly 5 weeks cumul	1848.	20 470.2	{20 366., 20 570.}	1007.69	1.04442	newly 55 weeks cumul	1919.	27 193.9	{27 102., 27 290.}	1317.09	1.1514
42	newly 3 weeks spec	1150.	4593.11	{4510., 4673.}	299.401	0.601367	newly 3 weeks cumul	5514.	16 068.2	{15 935., 16 188.}	191.406	0.464491	newly 5 weeks spec	480.	3196.2	{3122., 3269.}	565.875	0.823326	newly 5 weeks cumul	6798.	23 121.2	{22 984., 23 246.}	240.118	0.531625	newly 48 weeks cumul	7662.	36 279.9	{36 033., 36 497.}	373.505	0.67532
43	newly 3 weeks spec	1025.	4119.15	{3989., 4216.}	301.868	0.603984	newly 3 weeks cumul	4521.	14 296.9	{14 078., 14 507.}	216.233	0.49997	newly 5 weeks spec	563.	2922.74	{2815., 3020.}	419.137	0.715092	newly 5 weeks cumul	5852.	20 703.4	{20 402., 21 102.}	253.783	0.548681	newly 47 weeks cumul	6825.	33 246.3	{32 589., 33 960.}	387.125	0.687543
44	newly 3 weeks spec	487.	4395.23	{4310., 4470.}	802.511	0.955409	newly 3 weeks cumul	3260.	17 132.6	{17 020., 17 252.}	425.54	0.7206	newly 5 weeks spec	138.	2589.59	{2526., 2645.}	1776.51	1.27328	newly 5 weeks cumul	3654.	23 110.3	{22 992., 23 214.}	532.466	0.801034	newly 54 weeks cumul	3820.	31 012.5	{30 858., 31 143.}	711.845	0.90947
45	newly 3 weeks spec	960.	3969.28	{3857., 4089.}	313.467	0.616335	newly 3 weeks cumul	5111.	15 275.2	{15 064., 15 518.}	198.869	0.475443	newly 5 weeks spec	424.	2383.43	{2291., 2466.}	462.13	0.749655	newly 5 weeks cumul	6116.	20 737.9	{20 452., 21 045.}	239.075	0.530254	newly 52 weeks cumul	6740.	28 331.9	{27 801., 28 841.}	320.355	0.623543
46	newly 3 weeks spec	833.	5280.77	{5165., 5389.}	533.946	0.802004	newly 3 weeks cumul	4606.	18 393.9	{18 254., 18 498.}	299.346	0.601343	newly 5 weeks spec	254.	3581.22	{3497., 3665.}	1309.93	1.14913	newly 5 weeks cumul	5399.	26 376.2	{26 213., 26 537.}	388.538	0.688894	newly 51 weeks cumul	5711.	39 520.4	{39 264., 39 763.}	592.006	0.840104
47	newly 3 weeks spec	1599.	3395.84	{3208., 3585.}	112.373	0.326714	newly 3 weeks cumul	6760.	12 260.8	{11 797., 12 823.}	81.3729	0.258337	newly 5 weeks spec	834.	2278.93	{2141., 2423.}	173.253	0.43602	newly 5 weeks cumul	8781.	17 337.	{16 659., 18 128.}	97.4381	0.295167	newly 47 weeks cumul	10 888.	26 380.	{24 808., 27 911.}	142.285	0.383846
48	newly 3 weeks spec	1207.	3661.91	{3542., 3765.}	203.389	0.481839	newly 3 weeks cumul	5943.	14 235.1	{13 879., 14 507.}	139.528	0.379274	newly 5 weeks spec	486.	2175.32	{2076., 2295.}	347.597	0.650568	newly 5 weeks cumul	7196.	19 239.1	{18 701., 19 698.}	167.358	0.426998	newly 52 weeks cumul	7925.	26 058.4	{25 068., 26 836.}	228.813	0.516809
49	newly 3 weeks spec	149.	4994.2	{4909., 5072.}	3251.81	1.52524	newly 3 weeks cumul	1122.	19 354.6	{19 223., 19 473.}	1625.01	1.23679	newly 5 weeks spec	19.	2948.29	{2889., 3004.}	15 417.3	2.19076	newly 5 weeks cumul	1199.	26 151.7	{26 025., 26 286.}	2081.13	1.33868	newly 56 weeks cumul	1212.	35 070.2	{34 934., 35 208.}	2793.58	1.46143
50	newly 3 weeks spec	163.	3963.46	{3887., 4034.}	2331.57	1.38584	newly 3 weeks cumul	1147.	15 635.8	{15 509., 15 737.}	1263.19	1.13455	newly 5 weeks spec	19.	2298.46	{2228., 2356.}	11 997.2	2.08257	newly 5 weeks cumul	1229.	20 944.2	{20 833., 21 047.}	1604.17	1.23151	newly 55 weeks cumul	1262.	27 815.	{27 711., 27 915.}	2104.04	1.34322
